# The Health Informatics Trial Enhancement Project (HITE): Using routinely collected primary care data to identify potential participants for a depression trial

**DOI:** 10.1186/1745-6215-11-39

**Published:** 2010-04-15

**Authors:** Joanna McGregor, Caroline Brooks, Padmaja Chalasani, Jude Chukwuma, Hayley Hutchings, Ronan A Lyons, Keith Lloyd

**Affiliations:** 1 Centre for Health Information Research and Evaluation (CHIRAL), School of Medicine, Grove Building, Swansea University, Singleton Park, Swansea, UK

## Abstract

**Background:**

Recruitment to clinical trials can be challenging. We identified anonymous potential participants to an existing pragmatic randomised controlled depression trial to assess the feasibility of using routinely collected data to identify potential trial participants. We discuss the strengths and limitations of this approach, assess its potential value, report challenges and ethical issues encountered.

**Methods:**

Swansea University's Health Information Research Unit's Secure Anonymised Information Linkage (SAIL) database of routinely collected health records was interrogated, using Structured Query Language (SQL). Read codes were used to create an algorithm of inclusion/exclusion criteria with which to identify suitable anonymous participants. Two independent clinicians rated the eligibility of the potential participants' identified. Inter-rater reliability was assessed using the kappa statistic and inter-class correlation.

**Results:**

The study population (N = 37263) comprised all adults registered at five general practices in Swansea UK. Using the algorithm 867 anonymous potential participants were identified. The sensitivity and specificity results > 0.9 suggested a high degree of accuracy from the algorithm. The inter-rater reliability results indicated strong agreement between the confirming raters. The Intra Class Correlation Coefficient (Cronbach's Alpha) > 0.9, suggested excellent agreement and Kappa coefficient > 0.8; almost perfect agreement.

**Conclusions:**

This proof of concept study showed that routinely collected primary care data can be used to identify potential participants for a pragmatic randomised controlled trial of folate augmentation of antidepressant therapy for the treatment of depression. Further work will be needed to assess generalisability to other conditions and settings and the inclusion of this approach to support Electronic Enhanced Recruitment (EER).

## Background

Recruitment to clinical trials in primary care can be challenging [1]. Recent papers in Trials have reported a variety of strategies to improve trial recruitment [2-4]. Drawing on the expanding field of health informatics, we report on a strategy to identify potential trial participants using routinely collected anonymised data that complements other approaches to this question [5]. Virtually all general practices in the UK hold patient medical records in electronic format. This level of computerisation is in line with the NHS 1998 Information for Health Strategy's goal of full implementation of person-based Electronic Health Records (EHRs) at the primary care level by 2005. Routinely collected data are recorded in both narrative and structured formats. In the structured format, data are presented in codes. The coding system adopted by the Department of Health for general practice is the Read Terminology [6], although plans are underway to migrate to Systematised Nomenclature for Medicine - Clinical Terms (SNOMED CT) [6]; which has been selected as the standard terminology scheme for the NHS Care Records Service and for the National Programme for IT and will eventually replace the current Clinical (Read) codes. Large volumes of routinely collected data held in electronic format are becoming increasingly available. Improvements in data quality as well as technological advancement and expertise in retrieving, transporting, storing, linking and analysing these data is leading to Health Informatics emerging as a field rich with potential for research purposes [7].

Most randomised studies in general practice use conventional methods for patient selection, recruitment and data collection. One conventional method is through General Practitioner (GP) referral to research trials. Doctors normally recruit when patients present themselves at appointments. However a limitation with this method is that no referral will be made for those patients who do not attend their appointments, therefore little can be said about the generalisability of the data. Other recruitment strategies include manual searches through patient records or database searches using diagnostic criteria to select and recruit patients and then sending out participant information sheets. Further strategies include use of multi-media, such as the internet [5], newsletters and also mail shots. Further strategies include use of multi-media, such as the internet, newsletters and also mail shots. All searches for potential participants that involve disclosure of identifiable information (without patients' consent) are undertaken by the patients' direct healthcare team. However, if there is no other practicable alternative to conducting the research an application can be made to the National Information Governance Board (NIGB). NIGB oversees applications for the common law duty of confidentiality to be set aside in specific circumstances, in accordance with Section 251 of the NHS Act [8].

Many trials in primary care fail to achieve satisfactory levels of recruitment.

Difficulties with achieving the target recruitment populations within fixed timeframes were observed as common problems [9]. A number of barriers to clinician participation have been identified including time constraints, lack of staff and training, and concern about the impact on doctor-patient relationship [10,11]. In addition, barriers to GP referrals in depression trials have included the unsuitability of the content and style of depression consultations and the perceived intrusiveness of introducing research into a complex consultation [12]. It seems that the demands on patients and clinicians need to be kept to a minimum [10].

Routine data may overcome some of these issues. It may eliminate the need for doctors to identify suitable patients when they attend the practice. The significant advantage is that larger numbers of suitable patients can be identified by this method in a shorter period of time, thus maximising recruitment and minimising costs. However, it should be noted that routine data requires validation, which needs to be factored into the resource and economic planning.

The Health Information Research Unit (HIRU)[13] based in the School of Medicine, Swansea University has been formed to harness the potential of routinely collected data. HIRU has established the Secure Anonymised Information Linkage (SAIL) database, which is a vast data repository of anonymised person-level data, as provided by an expanding group of Data Providers [14]. In total so far, around 700 million records, pertaining to Health and Social care events have been loaded into the SAIL Data Bank. HIRU, in conjunction with Health Solution Wales, UK (HSW) have developed a robust anonymisation system to ensure confidentiality whilst making the data available for research [15,16].

The purpose of this study was to construct a methodology for identifying potential participants for a trial using the routinely collected data stored in the SAIL databank and to determine if the methodology could correctly identify potential participants for a clinical trial. The trial identified for this project is the FolATED study, which is a pragmatic randomised controlled trial of folate augmentation of antidepressant therapy in the treatment of depression. It is currently being conducted in Wales, UK [17].

### Aim

To determine whether anonymised routine data can be used to accurately identify the numbers of eligible patients suitable for recruitment to an existing randomised controlled trial (RCT).

### Objectives

• To construct an algorithm to identify suitable participants for a clinical trial using routinely collected, anonymised primary care data stored in the SAIL databank.

• To carry out a validation exercise to establish whether the algorithm could correctly identify potential participants.

## Methods

### Algorithm construction

The FolATED RCT inclusion and exclusion criteria and the timeframes for each criterion (see Tables 1 &2) were used as the basis to determine whether appropriate patients could be identified within the SAIL databank. These clinical criteria were translated into codified controlled measures (Read codes Version 2- [See Additional File 1]. The NHS Read Terminology Version 2 (5-byte) browser was used to identify appropriate read codes. To ensure that all exclusion criteria had been taken into account, the new GP Contract Qualities and Outcomes Framework (QOF) Version 10 Indicator Sets were used as an additional tool to identify codes for depression, learning disability, psychoses, Lithium prescription, palliative care and cancer. The British National Formulary (BNF) Version 54 [18] was checked for criteria relating to drugs and medicines, although all the relevant read codes were identified from the NHS browser, both by generic and brand names.

**Table 1:** Results of applying inclusion and exclusion criteria to identify potential trial participants from five general practices

Criterion applied	Potential participants remaining
Aged 18 years and over	37263
Antidepressant therapy within last 3 months	2650
Lifetime diagnosis of moderate to severe depression	1247
Folate deficient <2.5 ng/L at any time	1237
B12 deficient <150 pg/ml at any time	1196
Taking folic acid supplements in last 2 months	1180
Lifetime diagnosis of psychosis	1116
Pregnant or planning pregnancy in last 10 months	1116
Taking anticonvulsants at any time	1008
Life expectancy less than 1 year	1007
Any unstable medical condition in last 12 months	976
Taking Lithium at any time	965
Lifetime diagnosis of malignancy	873
Adverse reaction to folic acid a any time	873
Depression resolved in last 3 months	872
Lifetime diagnosis of delirium	871
Lifetime diagnosis of learning disability	867

**Final number of potential trial participants**	**867**

**Table 2:** Detection by algorithm of potential trial participants compared to detection by two independent clinicians.

Test characteristic	Clinician 1	Clinician 2
True Positives	87	81
False negatives	1	7
False Positives	0	3
True negatives	80	77
N	168	168

Sensitivity	0.92	0.99
Specificity	0.96	1.0
Positive predictive value	0.96	1.0
Negative predictive value	0.92	0.99

### Inclusion and Exclusion Criteria

Inclusion and exclusion criteria are summarised in Table 3. From the five practises we included all persons aged 18 and over. We then used recent antidepressant therapy (within last three months) as a proxy measure to identify a large enough sample of people who were currently suffering from depression. We then applied a diagnostic criterion of a diagnosis of moderate to severe depression within their medical history recorded on the SAIL databank (1993-2007). This latter criterion was intended to increase specificity and to reduce the possibility of capturing patients who suffered from mild depression, senile dementia with depression and other medical conditions. Finally we applied the FolATED study exclusion criteria to the algorithm in order to remove ineligible patients from the sample. Scoring on any one of these criteria led to exclusion.

**Table 3:** Trial inclusion and exclusion criteria used to search patient database

Inclusion criteria	Time Frame
Aged 18 years and over	At any time
And Antidepressant therapy	Within last 3 months
And Diagnosis of moderate to severe depression	At any time

**Patients excluded on basis of any one of**	

Folate deficient <2.5 ng/L	At any time
B12 deficient <150 pg/ml	At any time
Taking folic acid supplements	Within last two months
Past diagnosis of psychosis	At any time
Pregnant or planning pregnancy	Within last 10 months
Taking anticonvulsants	At any time
Life expectancy less than 1 year	At any time
Any unstable medical condition	Within last 12 months
Taking Lithium	At any time
Any malignancy	At any time
Adverse reaction to folic acid	At any time
Depression resolved	Within last three months
Delirium	At any time
Learning disability	At any time

### Data analysis

A database query using the Structured Query Language (SQL) was constructed using the identified read codes. The algorithm was run against the General Practice Database (GPD) within SAIL.

### Validation

Two samples were identified, in order to check the validity of this methodology for selecting potential participants. A 10% random sample of the eligible patients were chosen and a second sample of eighty ineligible patients was selected from the main SAIL database. The two samples were combined to form one dataset. Two independent mental health clinicians were given secure access to the anonymised health records of these selected patients and rated their eligibility for the trial. The sensitivity and specificity of the method of routine data capture to select eligible patients was calculated using clinical judgement of diagnosis as the 'gold standard'.

### Statistical analysis

Sensitivity and specificity tests were carried out to measure the reliability and accuracy of the results from the validation exercise, comparing the algorithm decision with the clinical judgement. Intra-class correlation coefficient and the kappa Statistic were carried out to measure the agreement between the two clinicians. Statistical analyses were performed using SPSS version 13 [19].

### Data anonymisation

HIRU has a protocol in place with HSW to ensure that all data is anonymised. This has been achieved through the split file approach to data management. The demographic data is separated from the clinical data by the source organisation and a system linking field is used to ensure that the data can be rejoined later. The demographic data is sent to HSW and the clinical data is sent to HIRU. HSW use encryption technology for pseudonymisation, replacing the personal data in each record with an Anonymous Linking Field (ALF). This product is then transferred to HIRU where it is joined to the clinical data via the system linking field. As a final safeguard HIRU further encrypts the ALF, thus ensuring that no single organisation can decrypt the records. This split file method ensures that anonymity and confidentiality is maintained, whilst maintaining the facility of data linkage at the individual level. The data is then ready for research applications [16]. Only the source organisation (i.e. the treating physician) has access to both personal and clinical data. The data is provided to the SAIL database on the grounds that it is never deanonymised, patient records can never be traced back to individual patients.

### Ethics

The work of the SAIL databank is conducted in strict accordance with a suite of data management policies which take account of the Data Protection Act (1998)[20], the Principles of the Caldicott report (1997)[21] and other measures that embody good practice in information governance. The information principles underpinning the work of SAIL have been endorsed by Informing Healthcare [22] and the Corporate Health Information Programme (CHIP)[23] and have been reviewed by Caldicott Guardians and Information Governance Officers in the NHS and Local Government. At a project level, all proposals for data utilisation are scrutinized for compliance with information governance by an independent panel comprised of members from: the British Medical Association, Informing Healthcare, Public Health Wales NHS Trust, Involving People and the Multi-centre Research Ethics Committee for Wales [14].

The FolATED study has been approved by the Multicentre Research Ethics Committee for Wales and by three Local Research Ethics Committees (North East Wales, North West Wales and Swansea Research).

## Results

### Identifying potential participants in SAIL

The practice data on the SAIL databank at the time of analysis included data up to 31/8/07. From the sample population of 37,263 patients aged 18 and over 2650 were identified as having been prescribed antidepressants within the last three months (7.11%). Of these 2650 patients 1247 had a diagnosis of moderate to severe depression within their medical history recorded on the SAIL databank (since ~1993). After application of all the inclusion and exclusion criteria 867 potential trial participants for the FolATED study were identified (Table 1).

### Validation

88 cases identified using the algorithm and 80 randomly selected individuals who did not meet the inclusion criteria were selected from the SAIL data bank. The accuracy of the algorithm was compared against the judgment of two psychiatrists (PC) and (JC) who independently rated all 168 records for evidence of a diagnosis of current depression. Table 2 shows the sensitivity and specificity of the algorithm against the clinical gold standard.

The two raters' independent classification of cases had an intra-Class Correlation Coefficient ICC (Cronbach's Alpha) of 0.93; (excellent agreement >= 0.9)[24,25] and a Cohen's Kappa Coefficient of 0.87; (almost perfect agreement >= 0.8) [24,25].

## Discussion

The algorithm for identifying suitable participants for the FolATED study appears to be valid based on the clinical judgment of the raters. The results from the sensitivity and specificity suggested a high degree of accuracy (>= 80%) from the algorithm. Although some minor methodological issues were encountered, we have demonstrated that it is possible to identify anonymous potential trial participants using the routinely collected primary care data.

### Limitations of the proposed method

A system based on anonymised data cannot be applied directly to recruitment strategies, as for instance the data housed in SAIL can never be deanonymised.

So this method we are exploring is a two part process. Firstly creating, testing and validating an algorithm to identify suitable participants using the anonymised data in SAIL. Then making this algorithm available on a live practice based computer based facility (such as Audit+ [26]) whereby a physician can run the query themselves and generate a list of suitable named participants within the practice, with minimal time or effort. Thus this should reduce GPs workload, with the potential of maximising recruitment. The method ensures confidentiality of personal data as the identification and recruitment process remains within the practices. This process itself requires validation. Furthermore, missing or implausible data values in the electronic records cannot be corrected as it is not possible to identify the patient.

Additionally, there was also an additional requirement for the researcher to seek clinical expertise to identify appropriate read codes. For example, medical advice was sought as to whether to include read codes relating to post viral depression and pre-senile dementia with depression in the algorithm.

### Limitations of routinely collected data

There are also a number of general limitations to the use of routinely collected data. The accuracy of using proxy measures needs to be evaluated. Lack of linkage between diagnosis and therapy makes the use of proxy measures unreliable. This issue is not limited to this methodology but applies to live database searches too. In this study recent antidepressant therapy was used as a proxy measure for depression to try to capture patients who were currently depressed, as the diagnosis may not be recorded as frequently as the treatment prescribed if it is an ongoing condition. The use of antidepressants as a proxy measure for depression is unreliable because disorders cannot be linked to specific interventions i.e. drugs [27]. An attempt to counter this was made by selecting people who had a diagnosis of moderate to severe depression in their medical history, however there was no way of knowing whether their current antidepressant therapy was related to that diagnosis. Antidepressant therapy may have been prescribed for other conditions, such as anxiety disorders, attention deficit disorder or dementia. It would be useful if there was a standardised 'problem number' field in all primary care data entry systems that linked the prescription to the diagnosis. The Meditel system has this field [28].

A particular challenge is establishing the end date of an episode of depression and whether or not the patient is in remission. The codes that might assist in identifying this, such as depression resolved, medication stopped and medication changed, may be infrequently employed and therefore cannot be relied upon as accurate measures in themselves.

Routinely collected data are captured for administrative reasons rather than for research purposes. To be fit for research purposes the validity, accuracy and completeness of the routine data itself need to be considered. Although studies have reported that routinely collected diagnostic data held on general practice information systems are accurate and reliable for research purposes [29-31], there is always room for initiatives to standardise systems and to improve data quality in primary care [32].

The purpose of this study was to model using anonymised data a new method of identifying suitable participants using routinely collected data that would make it easier for practices to identify potential subjects for a clinical trial and consequently reduce their workload, whilst potentially maximising recruitment and reducing costs. In the future we will seek to test this algorithm on clinical data sets within primary care settings. The algorithm that was created in this study successfully identified suitable anonymous participants for the trial within the SAIL environment. However the data within SAIL can never be deanonymised. Therefore the next phase is a pilot project for the translation of the algorithm running on anonymised SAIL data to run on live clinical systems, where the individual physician can generate a list of potential identifiable participants, with minimal time and effort. The method ensures confidentiality of personal data as the identification and recruitment process remains within the practices.

## Conclusions

The use of routinely collected digitally stored clinical data from primary care can be used as a means of selecting anonymous possible participants for a trial of folate augmentation of antidepressant therapy. Future work is required to run this algorithm on patient identifiable systems within the primary care practice setting and then compare this method with the traditional non-electronic method of participant identification for recruitment, in terms of numbers recruited, time, cost and reliability.

## Competing interests

The authors declare that they have no competing interests.

## Authors' contributions

KL and HH conceived the study and participated in its design and coordination and drafted the manuscript. RL contributed to the design. CB provided technical assistance in writing the SQL script. JM & KL carried out the analyses, performed the statistical analysis and drafted the manuscript. JC and PC were the independent clinicians who rated the eligibility of the patients identified in SAIL. All authors read and approved the final manuscript.

## Supplementary Material

Additional file 1 **Read Codes Version 2 (5-byte) for Folated Inclusion and exclusion criteria**. This file contains the NHS Read codes for the inclusion and exclusion criteria described in Table 3. Click here for file

## References

[B1] Vickers MR Martin J Meade TW The Women's international study of long-duration oestrogen after menopause (WISDOM): a randomised controlled trial BMC Womens Health 2007 7 2 10.1186/1472-6874-7-2 17324282 PMC1828722

[B2] Paine BJ Stocks NP Maclennan AH Seminars may increase recruitment to randomised controlled trials: lessons learned from WISDOM Trials 2008 9 5 10.1186/1745-6215-9-5 18226264 PMC2249567

[B3] Rahbari NN Diener MK Fischer L Wente MN Kienle P Buchler MW Seiler CM A concept for trial institutions focussing on randomised controlled trials in surgery Trials 2008 9 3 18218081 10.1186/1745-6215-9-3 PMC2259294

[B4] Dugas M Lange M Berdel WE Muller-Tidow C Workflow to improve patient recruitment for clinical trials within hospital information systems - a case-study Trials 2008 9 2 10.1186/1745-6215-9-2 18186949 PMC2253503

[B5] Brooks CJ Stephens JW Price DE Ford DV Lyons RA Prior SL Bain SC Use of a patient linked data warehouse to facilitate diabetes trial recruitment from primary care Prim Care Diabetes 2009 3 245 8 10.1016/j.pcd.2009.06.004 19604741

[B6] Connecting for Health: Systems and Services http://www.connectingforhealth.nhs.uk/systemsandservices/data/snomed

[B7] de Lusignan S van Weel C The use of routinely collected computer data for research in primary care: opportunities and challenges Fam Pract 2006 23 2 253 263 10.1093/fampra/cmi106 16368704

[B8] National Information Governance Board for Health and Social Care (NIGB) National Information Governance Board for Health and Social Care (NIGB) http://www.nigb.nhs.uk/

[B9] Campbell MK Snowdon C Francis D Elbourne D McDonald AM Knight R Entwistle V Garcia J Roberts I Grant A Grant A STEPS group Recruitment to randomised trials: strategies for trial enrolment and participation study. The STEPS study Health Technol Assess 2007 11 48 iii, ix-105 17999843 10.3310/hta11480

[B10] Prescott RJ Counsell CE Gillespie WJ Grant AM Russell IT Kiauka S Colthart IR Ross S Shepherd SM Russell D Factors that limit the quality, number and progress of randomised controlled trials Health Technol Assess 1999 3 20 1 143 10683591

[B11] Ross S Grant A Counsell C Gillespie W Russell I Prescott R Barriers to participation in randomised controlled trials: a systematic review J Clin Epidemiol 1999 52 12 1143 1156 10.1016/S0895-4356(99)00141-9 10580777

[B12] Mason VL Shaw A Wiles NJ Mulligan J Peters TJ Sharp D Lewis G GPs' experiences of primary care mental health research: a qualitative study of the barriers to recruitment Fam Pract 2007 24 5 518 525 10.1093/fampra/cmm047 17698979

[B13] Health Informatics Research Unit http://www.swan.ac.uk/ils/Research/CHIRAL/ResearchProgramme/HealthInformationResearchUnit/

[B14] Ford DV Jones KH Verplancke JP Lyons RA John G Brown G Brooks CJ Thompson S Bodger O Couch T Leake K The SAIL Databank: building a national architecture for e-health research and evaluation BMC Health Serv Res 2009 9 157 10.1186/1472-6963-9-157 19732426 PMC2744675

[B15] Health Solution Wales http://www.hsw.wales.nhs.uk/

[B16] Lyons RA Jones KH John G Brooks CJ Verplancke JP Ford DV Brown G Leake K The SAIL databank: linking multiple health and social care datasets BMC Med Inform Decis Mak 2009 9 3 10.1186/1472-6947-9-3 19149883 PMC2648953

[B17] Roberts SH Bedson E Hughes DA Lloyd KR Moat S Pirmohamed M Slegg GP Tranter R Whitaker R Wilkinson C Russell I Folate Augmentation of Treatment - Evaluation for Depression (FolATED): protocol of a randomised controlled trial BMC Psychiatry 2007 7 1 65 10.1186/1471-244X-7-65 18005429 PMC2238748

[B18] British National Formulary http://bnf.org/bnf/

[B19] George D Mallery P SPSS for Windows step by step: A simple guide And reference. Volume 11.0 update 2003 4 Boston: Allyn & Bacon

[B20] The Office of Public Sector Information (OPSI) The Data Protection Act (1998) http://www.opsi.gov.uk/Acts/Acts1998/ukpga_19980029_en_1

[B21] Department of Health: The Caldicott Report http://www.dh.gov.uk/en/Publicationsandstatistics/Publications/PublicationsPolicyAndGuidance/DH_4068403

[B22] Informing Healthcare: Welsh assembly government strategy to support the modernisation of health services using information and communication technologies http://www.wales.nhs.uk/ihc/

[B23] National Assembly for Wales: Corporate Health Information Programme (CHIP) http://www.assemblywales.org/

[B24] Landis JR Koch GG The measurement of observer agreement for categorical data Biometrics 1977 33 1 159 174 10.2307/2529310 843571

[B25] Shrout PE Fleiss JL Intraclass correlations: uses in assessing rater reliability Psychol Bull 1979 86 2 420 428 10.1037/0033-2909.86.2.420 18839484

[B26] Audit+ 25 http://www.informatica-systems.co.uk/audit-plus.aspx

[B27] de Lusignan S Valentin T Chan T Hague N Wood O van Vlymen J Dhoul N Problems with primary care data quality: osteoporosis as an exemplar Inform Prim Care 2004 12 3 147 156 15606987 10.14236/jhi.v12i3.120

[B28] Lawrenson R Williams T Farmer R Clinical information for research; the use of general practice databases J Public Health Med 1999 21 3 299 304 10.1093/pubmed/21.3.299 10528957

[B29] Anandarajah S Tai T de Lusignan S Stevens P O'Donoghue D Walker M Hilton S The validity of searching routinely collected general practice computer data to identify patients with chronic kidney disease (CKD): a manual review of 500 medical records Nephrol Dia Transplant 2005 20 10 2089 2096 10.1093/ndt/gfi006 16030033

[B30] Jick H Jick SS Derby LE Validation of information recorded on general practitioner based computerised data resource in the United Kingdom Bmj 1991 302 6779 766 768 10.1136/bmj.302.6779.766 2021768 PMC1669537

[B31] Nazareth I King M Haines A Rangel L Myers S Accuracy of diagnosis of psychosis on general practice computer system Bmj 1993 307 6895 32 34 10.1136/bmj.307.6895.32 8343670 PMC1678461

[B32] Roland M Linking physicians' pay to the quality of care - a major experiment in the United Kingdom N Engl J Med 2004 351 14 1448 1454 10.1056/NEJMhpr041294 15459308

