# Supplementary material for: The Health Informatics Trial Enhancement Project (HITE): Using routinely collected primary care data to identify potential participants for a depression trial
Source: Trials. 2010 Apr 15;11:39. doi: 10.1186/1745-6215-11-39 (PMC2864261; doi:10.1186/1745-6215-11-39)
Supplement: Additional file 1 — Read Codes Version 2 (5-byte) for Folated Inclusion and exclusion criteria. This file contains the NHS Read codes for the inclusion and exclusion criteria described in Table 3. [file 1745-6215-11-39-S1.DOC]

**Additional file 1: Read Codes Version 2 (5-byte) for Folated Inclusion and Exclusion Criteria**

**Antidepressant therapy**

d7% - Tricyclic Antidepressants

d8% - Monoamine-Oxidase Inhibitors

d9% - Compound Antidepressant drugs

da% - Other Antidepressant drugs

**Moderate to Severe Depression**

Eu321 - Moderate depressive episode

Eu322 - Severe depressive episode without psychotic symptoms

Eu331 - Recurrent depressive disorder, current episode moderate

Eu332 - Recurrent depressive disorder, current episode severe without psychotic symptoms

E1122 - Single major depressive episode, moderate

E1123 - Single major depressive episode, severe, without mention of psychosis

E1132 - Recurrent major depressive episodes, moderate

E1133 - Recurrent major depressive episodes, severe, without mention of psychosis

E2B1 - Chronic depression

E2B1. - Chronic depression

Eu32y - [X]Other depressive episodes

Eu32z - [X]Depressive episode, unspecified

Eu33y - [X]Other recurrent depressive disorders

Eu33z - [X]Recurrent depressive disorder, unspecified

E1137 - Recurrent depression

E291 - Prolonged depressive reaction

E112 - Single major depressive episode

E112. - Single major depressive episode

E113 - Recurrent major depressive episode

E113. - Recurrent major depressive episode

E2B - Depressive disorder NEC

E2B.. - Depressive disorder NEC

Eu32 - Depressive Episode

Eu32. - Depressive Episode

Eu33 - Recurrent depressive Disorder

Eu33. - Recurrent depressive Disorder

E112z - Single major episode NOS

E113z - Recurrent major depressive episode NOS

E1120 - Single major episode unspecified

E1130 - Recurrent major depressive episodes unspecified

Eu323 - Severe depressive episode with psychotic symptoms

Eu333 - Recurrent depressive disorder, current episode severe with psychotic symptoms

Eu251 - Schizoaffective disorder, depressive type

E1124 - Single major depressive episode, severe, with psychosis

E1134 - Recurrent major depressive episodes, severe, with psychosis

Eu204 - Post Schizophrenic depression

E130 - Reactive depressive psychosis

E135 - Agitated depression

Eu341 - Dysthymia

E1125 - Single major depressive episode, in partial or unspecified remission

E1135 - Recurrent major depressive episodes, in partial or unspecified remission

E11y2 - Atypical depressive disorder

Eu412 - [X]Mixed anxiety and depressive disorder

E2003 - Mixed anxiety and depressive disorder

**Folate Deficient (<2.5ng/L)**

D012. - Folate-deficiency anaemia

D0120 - Congenital folate malabsorption anaemia

D0121 - Folate-deficiency anaemia due to dietary causes

D0122 - Folate-deficiency anaemia, drug induced

D0123 - Folate-deficiency anaemia due to malabsorption

D0124 - Folate-deficiency anaemia due to liver disorders

D0125 - Macrocytic anaemia unspecified cause

D012z - Folate-deficiency anaemia NOS

42U2.- Serum folate low

42U8.- RBC folate low

42UB.- Whole blood folate low

C2620 - Folic acid deficiency

C304.- Disturbance of sulphur-bearing amino-acid metabolism

Dyu03 - [X]Other folate deficiency anaemias

42U3. - Serum folate borderline

42U9. - RBC folate borderline

42UC. - Whole blood folate borderline

**B12 deficiency <150pg/ml)**

C2621 - Vitamin B12 deficiency

D011. - Vitamin B12 deficiency anaemia

D0110 - Vitamin B12 deficiency anaemia due to dietary causes

D0111 - Vitamin B12 deficiency anaemia due to malabsorption with proteinuria

D011X - Vitamin B12 deficiency anaemia, unspecified

D011z - Other vitamin B12 deficiency anaemia NOS

Dyu02 - [X]Other vitamin B12 deficiency anaemias

D0130 - Combined B12 and folate deficiency anaemia

Dyu06 - [X]Vitamin B12 deficiency anaemia, unspecified

Dyu01 - [X]Other dietary vitamin B12 deficiency anaemia

F11x6 - Cerebral degeneration due to vitamin B12 deficiency

42T2. - Serum vitamin B12 low

42T3. - Serum vit B12 borderline

D0130 - Combined B12 and folate deficiency anaemia

D0112 - Transcobalamin II deficiency

7L185 - Intramuscular injection of Vitamin B12

66E% - B12 deficiency monitoring

**Folic Acid Supplementation**

i331. - FOLIC ACID 100microgram tablets

i332. - FOLIC ACID 5mg tablets

i333. - LEXPEC 2.5mg/5mL s/f syrup

i334. - FOLIC ACID 400micrograms tablets

i335.- PRECONCEIVE 400micrograms tablets

i336.- FOLIC ACID 2.5mg/5mL s/f syrup

i337.- CANTASSIUM FOLIC ACID 400micrograms tablets

i338. - FOLIC ACID 400micrograms/5mL sugar free oral solution

i339.- FOLICARE 400micrograms/5mL sugar free oral solution

8BP2. - Over the counter folic acid therapy

**Psychosis**

Eu323 - Severe depressive episode with psychotic symptoms

Eu333 - [X]Recurrent depressive disorder, current episode severe with psychotic symptoms

E1124 - Single major depressive episode, severe, with psychosis

E1134 - Recurrent major depressive episodes, severe, with psychosis

E11y. - Other and unspecified manic-depressive psychoses

E11y0 - Unspecified manic-depressive psychoses

E11y1 - Atypical manic disorder

E11y3 - Other mixed manic-depressive psychoses

E11yz - Other and unspecified manic-depressive psychoses NOS

E11z. - Other and unspecified affective psychoses

E11z0 - Unspecified affective psychoses NOS

E11zz - Other affective psychosis NOS

E13.. - Other nonorganic psychoses

E130. - Reactive depressive psychosis

E131. - Acute hysterical psychosis

E132. - Reactive confusion

E133. - Acute paranoid reaction

E134. - Psychogenic paranoid psychosis

E13y. - Other reactive psychoses

E13y0 - Psychogenic stupor

E13y1 - Brief reactive psychosis

E13yz - Other reactive psychoses NOS

E13z. - Nonorganic psychosis NOS

E1y.. - Other specified non-organic psychoses

E1z.. - Non-organic psychosis NOS

E00.. - Senile and presenile organic psychotic conditions

E01.. - Alchoholic psychoses

E0110 - Korsakov's alcoholic psychosis

E0111 - Korsakov's alcoholic psychosis with peripheral neuritis

E0112 - Wernicke-Korsakov syndrome

E01y. - Other alcoholic psychosis

E01yz - Other alcoholic psychosis NOS

E01z. - Alcoholic psychosis NOS

E02.. - Drug psychoses

E03.. - Transient organic psychoses

E03y3 - Unspecified puerperal psychosis

E04.. - Other chronic organic psychoses

Eu105 - [X]Alcoholic psychosis NOS

Eu106 - [X]Korsakov's psychosis, alcohol induced

E11.. - Affective psychoses

8HHs. - Referral to psychosis early intervention service

E1... - Non-organic psychoses

Eu30% - [X]Manic episode

Eu31% - [X]Bipolar affective disorder

E10% - Schizophrenic disorders

E110% - Manic disorder, single episode

E111% - Recurrent manic episodes

E114% - Bipolar affective disorder, currently manic

E115% - Bipolar affective disorder, currently depressed

E116% - Mixed bipolar affective disorder

E117% - Unspecified bipolar affective disorder

E12% - Paranoid states

Eu2% - {X]Schizophrenia, schizotypal and delusional disorders

**Pregnancy**

62… - Patient pregnant

621% - Patient currently pregnant

ZV22% - [V]Normal pregnancy

4654 - Urine pregnancy test positive

584E. - Antenatal ultrasound confirms ectopic pregnancy

584D. - Antenatal ultrasound confirms intra-uterine pregnancy

615C. - Pregnant, IUD failure

6174 - Pregnant, sheath failure

6166 - Pregnant, diaphragm failure

L03.. - Ectopic pregnancy

L032. - Ovarian pregnancy

13H7. - Unwanted pregnancy

L21.. - Multiple pregnancy

**Anticonvulsant therapy**

dn% - Control of Epilepsy

do% - Status Epilepticus drugs

8B66. - Anticonvulsant therapy

14P4. - H/O: anticonvulsant therapy

8BL3. - Patient on maximal tolerated anticonvulsant therapy

**Terminal Illness/Palliative care**

1Z0.. - Terminal illness

1Z00. - Terminal illness - early stage

1Z01. - Terminal illness - late stage

8H7L. - Refer for terminal care

8H6A. - Refer to terminal care consult

8BAN. - Community specialist palliative care

8BAP. - Specialist palliative care

8BAR. - Specialist palliative care treatment - inpatient

8BAS. - Specialist palliative care treatment - daycare

8BAT. - Specialist palliative care eatment - outpatient

8BJ1. - Palliative treatment

8BA2. - Terminal Care

8CM1. - On gold standards palliative care framework

8H7g. - Referral to palliative care service

8HH7. - Referred to community specialist palliative care team

9EB5. - DS 1500 Disability living allowance (terminal care) completed

ZV57C - [V]Palliative care

**Unstable medical condition**

212D. - Patients condition unstable

2127 - Patients condition worsened

**Lithium therapy**

d61% - Lithium Carbonate

d62% - Lithium Citrate

**Malignancy**

B% - Neoplasms (except B7% - Benign Tumour and ByuG% - (X)Benign

Neoplasms)

h% - Chemotherapy Immunosuppressant Drugs

**Folic Acid - Adverse Reaction/Poisoning/Abnormal**

TJ412 - Adverse reaction to folic acid

U6041 - [X] Adverse reaction to folic acid

SL410 - Folic acid poisoning

42UD. - RBC folate abnormal

**Depression resolved**

212S. - Depression resolved

**Delirium**

E030% - Delirium - acute organic

E031% - Delirium - subacute organic

Eu04% - [X]Delirium, not induced by alcohol and other psychoactive subs

E02y0 -Drug-induced delirium

E010. - Delirium tremens

E003. - Senile dementia with delirium

E0011 - Presenile dementia with delirium

E0041 - Arteriosclerotic dementia with delirium

Eu124 - [X]Mental and behavioural disorders due to use of cannabinoids:

withdrawal state with delirium

Eu164 - [X]Mental and behavioural disorders due to use of hallucinogens:

withdrawal state with delirium

Eu134 - [X]Mental and behavioural disorders due to use of sedatives or hypnotics: withdrawal state with delirium

Eu184 - [X]Mental and behavioural disorders due to use of volatile solvents:

withdrawal state with delirium

Eu104 - [X]Mental and behavioural disorders due to use of alcohol: withdrawal state with delirium

Eu144 - [X]Mental and behavioural disorders due to use of cocaine: withdrawal state with delirium

Eu114 - [X]Mental and behavioural disorders due to use of opioids: withdrawal state with delirium

Eu174 - [X]Mental and behavioural disorders due to use of tobacco: withdrawal state with delirium

Eu1A4 - [X]Mental and behavioural disorders due to use of crack cocaine:

withdrawal state with delirium

Eu194 - [X]Mental and behavioural disorders due to multiple drug use and use of other psychoactive substances: withdrawal state with delirium

Eu154 - [X]Mental and behavioural disorders due to use of other stimulants, including caffeine: withdrawal state with delirium

E042. - Chronic confusional state

Eu06Z - (X) Organic psychosyndrome

Eu03. - (X) Organic amnesic syndrome not induced alchohol/other psychoactive substances

G633. - Brain stem stroke syndrome

Eu107 - (X) Chronic alcoholic brain syndrome

2233. - O/E - delirious

**Learning Disabilities**

E3% - Mental retardation

Eu7% - [X]Mental retardation

Eu81z - [X]Developmental disorder of scholastic skills, unspecified

918e. - On learning disability register

**Key**

[X] – External causes of morbidity and mortality

[V] - Non illness reason for contact with health services

O/E – On examination

H/O – History of

NOS – Not otherwise specified
